# 2176. Cardiolipin Synthases Play Redundant Roles And Are Required for Membrane Remodeling in Daptomycin Resistant *Enterococcus faecalis*

**DOI:** 10.1093/ofid/ofad500.1798

**Published:** 2023-11-27

**Authors:** April Nguyen, Truc Tran, Diana Panesso, Kara S Hood, Vinathi Polamraju, Rutan Zhang, Ayesha Khan, William R Miller, Eugenia Mileykovskaya, Yousif Shamoo, Libin Xu, Heidi Vitrac, Cesar A Arias

**Affiliations:** University of Texas Health Science Center at Houston, Houston, Texas; Houston Methodist Research Institute, Houston, Texas; Houston Methodist Research Institute, Houston, Texas; Houston Methodist Research Institute, Houston, Texas; Washington University at St. Louis, St. Louis, Missouri; University of Washington, Seattle, Washington; Vanderbilt University, Nashville, Tennessee; Houston Methodist Research Institute, Houston, Texas; University of Texas Health Science Center at Houston, Houston, Texas; Rice University, Houston, Texas; University of Washington, Seattle, Washington; Mobilion Systems, Inc., Los Angeles, California; Houston Methodist and Weill Cornell Medical College, Houston, TX

## Abstract

**Background:**

Daptomycin (DAP) is a lipopeptide antibiotic targeting anionic phospholipids (APLs) at the division septum. Resistance to DAP (DAP-R) in *E. faecalis (Efs)* has been associated with activation of the LiaFSR response and redistribution of APL microdomains through the effector protein LiaX. We also identified a novel transmembrane protein (designated LiaY) as essential for changes in APL microdomains which contain cardiolipin (CL). *Efs* encodes two CL synthase genes, *cls1* and *cls2*. While changes in Cls1 are associated with DAP-R, their link to the LiaFSR system remains unclear. We aim to establish the roles of Cls in DAP-R to provide insights into membrane remodeling associated with DAP-R.

**Methods:**

qRT-PCR was used to study *cls* expression. Membrane lipid content was analyzed using hydrophilic interaction chromatography-mass spectrometry. Mutants were characterized by DAP minimum inhibitory concentration (MIC) using E-test and localization of APL microdomains with 10-N-nonyl-acridine orange. The bacterial two hybrid system was used to evaluate protein interaction. Fluorescence microscopy evaluated protein co-localization.

**Results:**

*cls1* was upregulated in DAP-R *Efs* OG117Δ*liaX*. Deletion of either *cls* caused upregulation of the remaining *cls* gene. Development of DAP-R altered membrane lipid content, namely, an increase in CL and a shift towards species with longer fatty acid chains and higher degree of unsaturation. Upon deletion of both *cls*, CL content was eliminated, peripheral APL microdomains were abolished, and a decrease in the DAP MIC was observed. LiaY interacted with Cls1, but not Cls2 using the two-hybrid system. The interaction was confirmed with fluorescence microscopy showing co-localization of LiaY and Cls1 at the septum and away from the septum in DAP-S and DAP-R strains, respectively. LiaY and Cls1 were also individually co-localized to APL microdomains.

**Conclusion:**

While Cls1 is predominantly associated with DAP-R, we show a functional redundancy between Cls1 and Cls2 in both membrane homeostasis and in DAP-R. Cls1 interacts with LiaY, which bridges the resistance mechanism with membrane adaptation, ultimately leading to altered localization of APL microdomains that divert DAP away from the division septum.

**Disclosures:**

**William R. Miller, M.D.**, Merck: Grant/Research Support|UpToDate: Honoraria

